# Detection of chicken DNA in commercial dog foods

**DOI:** 10.1186/s12917-022-03200-z

**Published:** 2022-03-09

**Authors:** Wioletta Biel, Małgorzata Natonek-Wiśniewska, Jagoda Kępińska-Pacelik, Katarzyna Kazimierska, Ewa Czerniawska-Piątkowska, Piotr Krzyścin

**Affiliations:** 1 Department of Monogastric Animal Sciences, Division of Animal Nutrition and Food, West Pomeranian University of Technology in Szczecin, 29 Klemensa Janickiego, 71-270 Szczecin, Poland; 2 Department of Animal Molecular Biology, National Research Institute of Animal Production, 1, Krakowska Street, 32-083 Balice, Poland; 3 Department of Ruminant Science, West Pomeranian University of Technology in Szczecin, Klemensa Janickiego 29, 71-270 Szczecin, Poland

**Keywords:** Adverse food reactions, Chicken DNA, Extruded dog food, Wet dog food, Pet food control, Ingredients, Mislabeling, Polymerase chain reaction

## Abstract

**Background:**

These days the number of potential food allergens is very large, but chicken is one of the most common allergens in dogs. Elimination diet is one of the clinical tools for the diagnosis of allergies and allergy tests are not very reliable. The restriction diet is most commonly carried out by feeding pet foods, relying on the ingredients on the label to select an elimination diet not containing previously eaten foods. Unfortunately, mislabeling of pet food is quite common. The purpose of this study was to determine the absence or presence of chicken DNA using both qualitative and quantitative polymerase chain reaction (PCR) analysis methods in dry and wet maintenance complete pet foods for adult dogs. Results were used to verify the declared composition on the labels.

**Results:**

Eleven out of fifteen (73%) dog foods were produced as declared by the manufacturer, two of which showed the presence of chicken protein as stated on the label. The remaining nine foods contained amounts of chicken DNA below 1%, consistent with declarations that no chicken was added in the composition. Four of tested dog foods (27%) were not produced consistently with the declaration on the packaging. Two dog foods (one dry and one wet) did not contain the claimed chicken protein. In two foods the addition of chicken DNA was detected at the level of over 2% and almost 6%, respectively.

**Conclusions:**

In this study, we focused on one of the most commonly undeclared animal species on the label—chicken protein—and performed DNA analyzes to investigate possible contamination and mislabeling. The results showed some inaccuracies. However, most of them are trace amounts below 1%, which proves compliance with the label. Our results showed that undeclared animal species can be as common as missing an animal protein declared on the label. The conducted research indicates that both dry and wet analyzed foods should not be recommended as a diagnostic tool in elimination tests, because it may result in false negative results. Over-the-counter maintenance foods for dogs should not be recommended for the diagnosis and treatment of food hypersensitivity.

**Supplementary Information:**

The online version contains supplementary material available at 10.1186/s12917-022-03200-z.

## Background

Food is a key element necessary to meet the basic needs of both humans and animals. That is why food quality and safety are so important and are constantly of interest to food analysts. A changing environment is forcing the food sector to modify food trends and change food consumption practices [1]. Nowadays, all consumer trends are moving towards healthier and more natural food options. Population growth generates an increasing demand for food, which leads to an increase in the production of processed food [2]. Therefore, governmental and official agencies must enforce guaranteed compliance with food labeling, nutritional quality and food origin, as well as the perception of health and diet claims in order to avoid false declarations of food producers. Therefore, the authenticity of food plays a significant role in food analysis.

Currently, the pet food market is constantly developing, but the most popular are maintenance foods, referred to as over-the-counter diets (OTC). Chicken-based ingredients are commonly used in pet food products. They are highly palatable, relatively inexpensive and provide an excellent source of protein, although individual products vary greatly in nutrient composition and processing conditions that may affect their protein quality and digestibility [3]. Dog caregivers are interested in OTC foods with limited protein sources because of their easy availability and relatively lower cost compared to veterinary therapeutic diets (VDT). For most dogs, chicken meat is easy to digest but its widespread usage in dog foods has likely led to more common allergies to this ingredient [4, 5]. Nowadays the number of potential food allergens is very large, but chicken is one of the most common allergens in dogs, after beef and dairy products [6]. Therefore, more and more dogs’ caregivers are avoiding pet food with added chicken for the fear of adverse reactions. Skin diseases are one of the most frequent presenting ailments for cats and dogs in veterinary practice [7, 8] and they are frequently caused by adverse food reactions (AFR). AFR is a common problem that may cause cutaneous and/or gastrointestinal signs in dogs and cats, including food intolerance, food intoxication and food allergy [9]. Neither in dogs nor in cats it is possible to distinguish food allergy (FA) from atopic dermatitis (AD). In the case of FA (which is an etiological diagnosis), it is possible to develop AD (which is a clinical diagnosis) [10]. Diagnosis of these diseases is difficult because AD and FA share common symptoms such as self-injury leading to erythema, alopecia, secondary infections, lichenification. However, there is often a coexistence of food allergy and atopic dermatitis in one individual [11, 12]. Dogs with AD have elevated levels of antibodies to food allergens (although an increase in these antibodies is not the same as food allergy) [13]. Establishing a reliable diagnosis of FA is only possible on the basis of an elimination diet and then a provocation test with food suspected of causing symptoms. The restriction diet is most commonly carried out by feeding dogs, relying on the ingredients on the label to select an elimination diet not containing previously eaten foods [14]. However, this process is time-consuming and heavily dependent on caregiver compliance and, apparently, on the integrity and reliability of pet food manufacturers [15]. According to an extensive review of the literature written by Olivry and Mueller [14], mislabeling of pet food is quite common.

The information provided on the dog food label should be comprehensive and include detailed information. Manufacturers, by law, must indicate on the label either all ingredients or categories, in a transparent, consistent, and understandable way [16]. Although the law and European Pet Food Industry Federation (FEDIAF) [17] stated that labels must be accurate and provide complete information on the ingredients, mislabeling of pet food has been documented by several authors. Already in the early twentieth century, species-specific ingredients in dog food were identified [18, 19], in which from thirty-one pet foods tested more than 30% dog foods contained ingredients not declared on the label. In a study conducted in Italy, considering the problem of contamination and mislabeling in dry and wet novel protein diets (NPDs) and hydrolyzed protein diets (HPDs), Ricci et al. [20] observed that out of forty analyzed products only ten (25%) presented a content correctly matched to the label. Many products were mislabeled, in which five did not contain the declared animal species and twenty-three revealed the presence of undeclared animal species. Moreover, it was found that 13 out of 14 brands tested, presented at least one mislabeled product. In a previous study by Ricci et al. [4], percentage of tested diets with detected ingredients not declared on the label exceeded 80% (ten out of twelve foods tested). Thus, discrepancies between ingredients and labeling seem to be alarmingly common for many years.

Therefore, the purpose of this study was to determine the absence or presence of chicken DNA using both qualitative and quantitative polymerase chain reaction (PCR) analysis methods, in dry and wet maintenance commercial complete foods for adult dogs with verifying the correctness of the composition on the label.

## Results

### DNA isolation and determinate standard curve

The concentration of DNA isolated from the analyzed samples was in the range 79.4–556.8 ng/µl (Table S2). The working range of repeatability for independent DNA isolation concentrations of the same dog food was in the range of 0.83–1.19% (Table S2). The purity of the concentrations expressed by the A_260/280_ parameter took values from 1.83 to 1.99 (Table S2) and their difference for repetitions did not exceed 5%. Working range of repeatability and little difference between absorbance ratio (< 5.0% higher value of absorbance ratio) (Table S2) allow to conclude that the applied isolation was performed with the appropriate accuracy and precision.

DNA isolated from chicken meat, which was used to generate the standard curve, reacted specifically in quantitative PCR. The shift coefficient was 21.2, the slope was -3.308, and the regression coefficient was 0.999. These parameters clearly indicate the biological specificity and linearity of the curve used. Such determined standard curve made it possible to accurately determine the potential content of chicken DNA. Additionally, the correct course of the reaction was confirmed by the results for both negative (NC) and positive controls (PC), which were determinate as 0.00 and 0.40%, respectively.

### Determination of chicken DNA

#### Dry foods

**Fig. 1:**
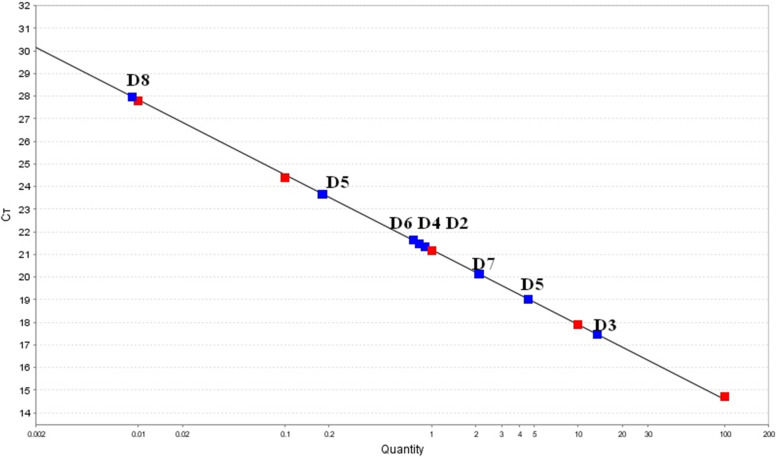
Calibration curves for evaluated dry dog food. Red points—points belonging to standard curve made of DNA isolated from chicken meat; Blue points—points belonging to researched dry dog foods; Quantity—concentration % chicken ingredients in researched samples (dry food); cT- amplification cut-off cycle

The results obtained for the analyzed dog foods are shown in Fig. 1, Table 1. These results are reproducible, the RSD for the average concentration was below 17.14% for food D1 and in most cases below 10% (foods D2, D4, D6, D7). The obtained results in most cases confirmed the manufacturer's declaration of chicken protein on the label. Chicken DNA was identified in foods D1 and D3, which was consistent with the manufacturer's declaration. However, the obtained results cannot be compared with the producer's declaration, because the quantitative composition was not given for the D1 feed, while for the D3 feed only the amount of poultry liver (4.5%) was given (Table 2). The obtained value of 13.65% may be proof of the declaration in accordance with reality. The results for D4 and D7 foods may be most questionable. Food D4 showed a content below 1% although the manufacturer declared the chicken protein presence, while D7 contained more than 2% chicken protein, which should be declared, and the information on the packaging does not contain any details about it. In the other foods, quantities of less than 1% or almost 0% (D8) were identified. Such a small amount is acceptable as an unintended artifact from the production line. In twelve tested dog foods (80%) the amount of chicken DNA was detected.

**Table 1:** Concentrations of chicken DNA in tested foods

Item	Cт Mean	Cт SD	Quantity Mean	Quantity SD	RSD%
D1	18.87	0.24	5.15	0.88	17.14
D2	21.38	0.13	0.89	0.08	9.08
D3	17.46	0.16	13.65	1.50	11.01
D4	21.42	0.05	0.87	0.03	3.83
D5	23.67	0.19	0.18	0.02	13.14
D6	21.55	0.07	0.79	0.04	5.08
D7	20.19	0.05	2.04	0.07	3.24
D8	27.68	0.24	0.01	0.00	
W1	27.07	0.17	0.02	0.00	
W2	29.80	0.08	0.00	0.00	
W3	34.84	1.39	0.00	0.00	
W4	28.49	0.34	0.01	0.00	
W5	30.49	0.15	0.00	0.00	
W6	18.65	0.13	5.96	0.54	8.98
W7	21.91	2.81	0.20	0.03	16.62
NTC	36.12	0.14	0.00	0.00	
PTC	22.54	0.01	0.40	0.00	

Cт Mean – average of Cт, where Cт – threshold cycle, Cт SD – Cт standard deviation, Quantity Mean—average chicken DNA concentration value for three independent DNA isolations, Quantity SD—average standard deviation of chicken DNA concentration for three independent DNA isolations, RSD %—relative standard deviation of chicken DNA concentration for three independent DNA isolations, *NTC* negative control, *PTC* positive control

**Table 2:** Marketing claims and ingredients containing or potentially containing chicken DNA declared on product labels

Item	Claim	Ingredient
D1	-	hydrolyzed chicken protein
D2	without grains and chicken	-
D3	-	animal fat (poultry 8%), hydrolyzed chicken protein (chicken liver hydrolysate 4.5%)
D4	-	animal fat (poultry), hydrolyzed chicken protein
D5	Hypoallergenic	-
D6	-	-
D7	-	-
D8	mono protein	-
W1	-	-
W2	-	-
W3	mono protein	-
W4	mono protein	-
W5	-	eggs 3%
W6	mono protein	-
W7	mono protein	-

#### Wet foods

**Fig. 2:**
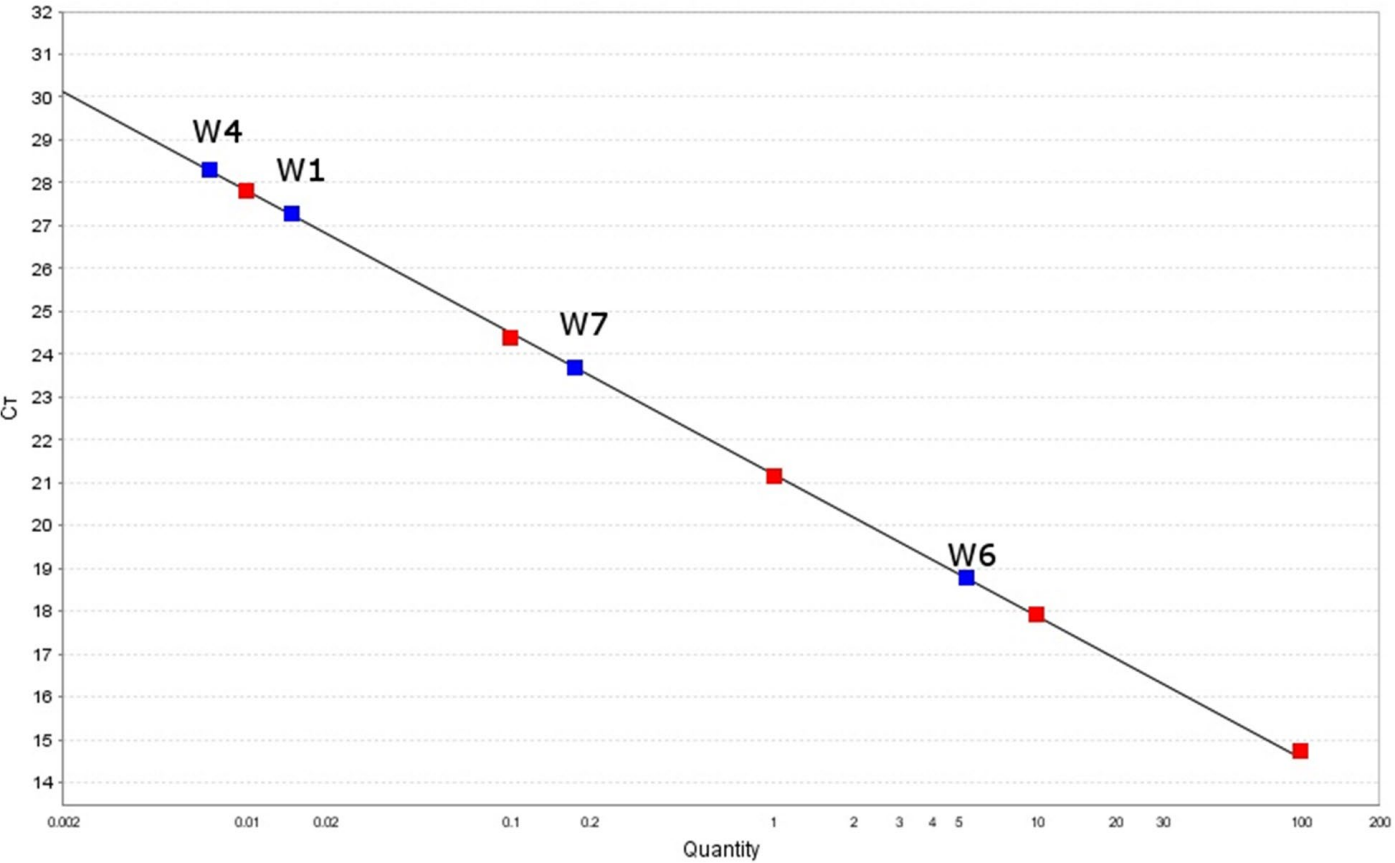
Calibration curves for evaluated wet dog food. Red points—points belonging to standard curve made of DNA isolated from chicken meat; Blue points—points belonging to researched wet dog foods; Quantity—concentration % chicken ingredients in researched samples (wet food); cT- amplification cut-off cycle

For most of the tested wet foods (W1-W4 and W7) (Fig. 2, Table 1) no chicken DNA was identified, which was consistent with the declaration on the package. Although traces of less than 0.02% were identified for the foods W1, W4, W7, this rather indicates the presence of artifacts from the production line than a conscious or intentional addition of the poultry component. For one of the tested foods (W5) no chicken DNA addition was detected although the eggs supplement was declared on the label. The manufacturer claimed that the dog food contained 3% eggs, but the analysis ruled out such an addition. The opposite situation was observed for the W6 food, in which it was identified an almost 6% addition of chicken DNA, despite the manufacturer's declaration that the food contains only protein from cattle (Table 2). For this food the result was repetitive, RSD was less than 9%.

## Discussion

Food safety is an extremely important issue related to human and animal health. Both raw materials and food ingredients must be constantly monitored, because some of the substances present in food are pollutants of anthropological origin, while others arise as a result of food processing or storage. Unfortunately, food counterfeiting is a common phenomenon. It can cause unfair competition, but also a feeling of cheating on the part of the consumer [21]. It also reduces the quality of food and may even pose a health risk. Both consumers and industry are asking the research community to implement tests that can help analyze food for authenticity.

Mentioned above problems are forcing food safety and quality scientists to constantly search for new approaches and tools to address the main current problems with food quality, safety and authenticity [22].

In recent years, many techniques have been developed to allow food traceability through DNA barcoding techniques even for complex and highly processed mixtures such as pet food. These techniques can identify adulteration in pet food products, which are an increasing problem among consumers and underpin several lawsuits in recent years [23, 24]. Properly selected methods can also provide a way to confirm that pet food is indeed "grain free", "corn free" or "chicken free" for example, claims that are becoming more common in many commercial pet products [25].

Considering the importance of precise pet food labelling, especially for animals with mild or life-threatening allergies, accurate detection of mislabeled or undeclared animal species is important for the safety of pets [23]. What is more, large variation among commercially available diets marketed for support the treatment of various medical conditions, such as skin and coat health or allergies, may cause confusion for caregivers during diet selection [26]. One of the problems with pet food mislabeling is the ingredient listing itself. Often, general or intentionally unclear ingredients are given [27]. Commission Regulation No. 767/09 allows the listing of ingredients by categories in pet foods [16].

Even despite existing mandatory traceability requirements for pet food, it has previously been reported that labels do not provide enough information on the sources of the various nutrients in the product. It is worth to borne in mind that pork, poultry and beef are the most commonly slaughtered animal species in Europe for human consumption [28]. Thus, they provide the largest amount of animal by-products to pet food producers. Consequently, the most common animal species not declared on the pet food labels seem to be pork, chicken and turkey. In one of the most extensive studies (number of foods tested = 52), chicken was even the most common meat species found in almost all (98%) tested pet food products [29].

Currently, there are several different methods of detecting protein in food and feed materials. For example, there is a study that determined the amount of herbicide (glyphosate) residues in commercial animal feed using an enzyme-linked immunosorbent assay (ELISA), in which each product tested contained detectable glyphosate residues [30]. However, Hsieh et al. [31] showed that ELISA-based methods appear to provide lower sensitivity and accuracy and were unable to fully identify the presence of animal by-products compared to PCR in commercial canine foods. At this time, PCR is as an official method of the determination of the ingredients of animal origin in animal food [32].

In this study, we also focused on one of the most common undeclared animal species on the label – chicken protein – and we performed DNA analyses on a total of 15 products to investigate possible contaminants and mislabeling. The results showed some inaccuracies. However, most of them were trace amounts below 1%, which indicates compliance with the label (D2, D5, D6, D8, W1, W4, W7). Two dry foods declared chicken protein added on the label (foods D1 and D3), which was confirmed by our research. In one of the tested dry foods (D4), chicken DNA was specified in the amount below 1%, despite the fact that the manufacturer declared the addition of chicken protein on the packaging. Setting the cut-off point to differentiate negative from positive samples at 1% has own source in the EURL-AP [33] in methods of the identification of meat components. In the reference methods, all identified components below 1% are considered an artifact. According to the information in Table 2, this food contains poultry fat and hydrolyzed protein. The term “animal fat” covers chicken fat, which may contain traces of this species' protein. It would have to be purified so as not to cause an allergic reaction. It happens that it is mentioned on the label, but not often [34, 35].

Food allergies are immunological reactions to food mediated by IgE (IgE-mediated) antibodies (type I hypersensitivity), but there are also IgE independent (non-IgE-mediated, T-cell dependent) (type II, III, IV) reactions [35]. Hydrolyzed proteins in commercial dog diets have a molecular weight of less than 10 kDa, and even less than 5 kDa. Generally, the theory behind the use of hydrolyzed diets in FA is that if proteins are split into molecular weights below 4–5 kDa, they will be too small to bridge two molecules of IgE on mast cells required to trigger the allergic reaction in IgE-mediated allergic diseases. At the same time, the enzymatic breakdown of proteins alone does not change their biological value [36]. While hydrolyzed diets have been shown to be useful in the diagnosis of dogs with suspected AFRs, most of these studies do not address the question of whether hydrolyzed diets control clinical signs in dogs hypersensitive to the parent protein. It is also unknown whether AFRs in dogs are necessarily IgE mediated [37]. In the dog, peptides with a molecular weight (MW) higher than 4.5 kDa could still be capable of starting the immunologic reaction which contributes to the allergic reaction [38, 39]. The process of protein treatment in order to create a hydrolyzed feed, unfortunately, is more and more often insufficient and does not guarantee the reduction of allergy to this protein, and even more so, it may be ineffective in the case of food allergy with non-IgE mediated reactions [34, 35, 38, 40, 41]. Hydrolyzed diets may still contain proteins that stimulate helpful T-cells responsible for regulating the immune response and may therefore be ineffective in treating hypersensitivity in animals. The same applies to an anallergenic diet (peptides below 1 kDa). As many as 40% of dogs show allergic reactions to proteins of even this MW [39, 42]. The above information is important because although hydrolyzed protein can also cause allergies, it is often incorrectly recommended for feeding dogs with food allergies.

A similar situation as in the case of dry food took place in the case of wet food (W5), where the declaration of the addition of 3% eggs on the packaging was not reflected in the actual amount of chicken DNA (0%). Chicken DNA was not found in this food (W5), but it should be borne in mind that egg (both whole egg and egg white, egg yolk) also causes allergies in dogs [43–45]. Jeffers et al. [46] conducted a study on thirteen dogs with food allergies to test the effectiveness of feeding with commercially available egg and rice food. They described a case of a dog with reaction to eggs, which in response to provocation ate dry diet with impunity, whereas another dog with severe pruritus while consuming the dry diet was not allergic to rice and hard-boiled eggs in response to dietary provocation. This collectively indicates that the processing of food may either partially or totally alter antigenicity, at least of eggs, or that the concentration of antigen may have an important role [46].

On the other hand, in foods D7 and W6, DNA levels above 2% were detected, despite the declaration of a single non-poultry protein source in the food. Our results showed that undeclared animal species could be as common as the lack of animal protein declared on the label. The studies of Dunham-Cheatham et al. [25] have shown that adulteration is common in commercial pet food. This fact is confirmed by the analyzes of Palumbo et al. [24] who found that 16 of 18 commercial pet foods tested were adulterated. Both studies have shown that ingredients with higher economic value (e. g. fish) are often supplemented or completely replaced with ingredients with lower economic value (e. g. chicken). In our research, less than 1% chicken DNA was found in fish feed. Detailed ingredients present on the tested dog food according to label information are presented in Table S1.

Such results lead to concern that consumers are paying unfairly prices for products that purportedly contain high-value ingredients, but in fact contain low-value ingredients. The incidence of adulteration in commercial pet food is also of concern for animals with food allergies. If the consumer cannot trust that pet food is free of these allergens, despite the label on the package, pets may still be exposed to hidden allergens and confidence in the pet food industry is seriously declining [25].

Similar results to our own research were obtained in previous studies. Hołda et al. [47] verified the potential presence of undeclared chicken-derived ingredients in ten commercially available dry dog foods using PCR technology and in most of the analyzed samples the chicken DNA was detectable. However, quantified amounts were mostly low (except one dog food with relatively high amounts of undeclared DNA) and the authors speculated that poor quality poultry fat might been used in the production of tested foods, most likely contaminated with proteins. Multiplex PCR was also used to screen the DNA of 10 mammalian species in a study to determine proteins in commercial dry dog foods with uncommon and limited ingredients [5]. The presence of DNA from one or more non-labeled species was identified in all 21 diets. However, no quantification of the contamination was determined, which precludes the determination of whether the addition of undeclared proteins was intentional or unavoidable cross-contamination. Similar studies were conducted on wet foods. Out of eleven canine and feline dietetic limited-antigen wet foods, analyzed by PCR, in six foods the contamination was confirmed [48].

Research by Horvath-Ungerboeck et al. [49] confirmed that the presence of undeclared protein on the label is quite common. In this study, nine out of ten OTC foods identified the DNA of one or more animal species other than those declared on the label. The most commonly detected DNA was from beef and pork. OTC “single protein” diets or wet foods should not be recommended for the diagnosis of dogs with AFR, as contamination may cause an elimination diet to fail.

The use of maintenance foods (OTC) as an elimination diet may be ineffective and result in false-negative results. OTC “single protein” diets should not be recommended for the diagnosis and treatment of food hypersensitivity.

According to Cox et al. [50], raw meat-based diets (RMBDs) are becoming more and more popular in the market, gaining acceptance by pet caregivers despite the potential health risks. In studies by Cox et al. [50], 9 commercial canine and 9 feline RMBDs were assessed for the presence of species-specific DNA. DNA from one or more unlisted animal species was identified in over 60% of diets. The most commonly detected, unlisted DNA was lamb in the diet of dogs and turkey in the diet of cats. Based on Cox et al. [50] results, the use of commercially available RMBD cannot be recommended as an elimination diet in the clinical diagnosis of AFR.

Undeclared animal proteins have also been detected in commercial vegetarian diets for dogs and cats. Out of fourteen commercially available dry and canned canine and feline diets marketed as vegetarian or vegan, seven (50%) were positive for one or more undeclared mammalian DNA source (bovine, porcine, or ovine) [51]. On the other hand, further studies examining prescription vegetarian diets for dogs for 11 species of mammals and poultry found that the food did not contain undeclared mammalian or avian proteins [52].

Vegan food producers declare that the food does not contain any animal-derived materials. However, such information must be verified by food control authorities and laboratory controls. The method used to detect trace amounts of animal-derived materials must be sensitive and enable the detection of DNA after processing and storage. These controls are designed to protect the consumer from fraud or mislabeling. European Union guidelines set a limit for unintended traces of animal residues to less than 0.1% (1 g/kg) of animal components [53, 54]. In the research by Köppel et al. [55], animal and fish DNA was detected in two products. One was found to contain cheese and egg, it was a vegetarian product, not a vegan product. The second showed a weak signal for the fish DNA. These studies suggest that further research is needed to move from qualitative screening to quantifying contamination in animals. Moreover, highly processed products, such as gelatin, eggs, and some ripened cheeses, do not contain enough DNA to exclude the presence of mammalian or fish residues in the products [55].

## Conclusions

The results of this study suggest that mislabeling occurs in various ways. It can refer to the lack of the declared ingredient on the label or conversely—the lack of the declaration of the added ingredient. It applies to both dry and wet food.

In the era of unprecedented DNA sequencing technology, the relative degree of mislabeling can be assessed by examining a wide range of pet food products for animal protein sources. The problem of discrepancies between ingredients and labeling still seems to be unsolved.

Accordingly, manufacturers are advised to systematically carry out stringent quality control. The aim of the controls should be to minimize cross-contact between components. They should also pay more attention to changed labeling information to increase consumer confidence when choosing food for their pets. The results of the research made it possible to verify the assumptions of our research. They can be the starting point for further research into the safety of commercial pet food.

## Methods

### Samples

The inclusion criteria for the diets selected for this study were that they had to be commercially available, popular and commonly available, dry or wet canine diets obtained from local pet stores and from online stores. All pet food were bought at pet stores. All of the selected products belonged to international brands and can be found in other European countries. Popularity was determined based on the sales information of pet stores.

Three packages of dry / canned (wet) of each pet food were purchased with different batch numbers. After opening, from three packages of individual dog foods samples were collected, in order to obtain one representative sample for chemical analyzes. The size of the packages ranged from 500 g to 2 kg (dry extruded food) and 400 g to 800 g (wet food).

In all, the research material consisted of fifteen complete maintenance diets for adult dogs, of which eight were dry extruded foods (D1-D8) and seven wet foods (W1-W7) (Table 2, Table S1). Dog foods were selected based on database of all products available on the local market, taking into account both foods that declare the addition of chicken protein on the ingredients list and foods that declared no chicken added. Pet foods labels were carefully read to identify every source of protein and fat in the ingredients list. Key nutritional information provided on the label was recorded such as ingredients list, macronutrient content (percentage protein, fat, moisture, ash and fiber, as fed) alongside the country of origin and batch number. The marketing claims and ingredients containing or potentially containing chicken DNA declared, on product labels of analyzed dog foods is shown in Table 2.

All analyzed dry and wet foods were packaged in sealed bags. Each analyzed feed was homogenized and made into a representative sample, and then three laboratory samples, each weighing 100 mg, were weighed. Between the homogenization of the samples, the grinding device was sterilized to avoid contamination.

### DNA identification

All food samples (D1-D8, W1-W7) were tested in triplicate. Total DNA was obtained with the AX Food kit (A&A Biotechnology, Gdynia, Poland), using the isolation methodology provided by the manufacturer, finally suspending the DNA in 50 µl of TE buffer. The DNA concentration was measured with a NanoDrop spectrophotometer (ND 2000; NanoDrop Technologies, Wilmington, DE, USA) while determining the purity of the extracted DNA by measuring the A_260/280_ absorbance ratio. The repeatability of the obtained isolates, and thus their suitability for further analysis, was determined by comparing the mass of the analyzed sample, the resulting DNA concentration and DNA purity for all three replicates of a given sample. In numerical terms, the working range of repeatability (Wr) was determined with the following formula:

1 $$\mathrm{Wr}\hspace{0.17em}=\frac{{c}_{1}{m}_{2} }{{c}_{2}{m}_{1}}$$

where:

m_1_—weight of first repeat of sample;

c_1_—the concentration of DNA obtained from it;

$\frac{{m}_{2}}{{c}_{2}}$—the same parameters obtained for the next repetition.

Determination of the amount of chicken DNA was performed at Step One Plus™ Real-Time PCR v2.3 (Thermo Fisher Scientific, MA USA). Primers complementary to the fragment encoding 16 SrRNA in chickens and TaqMan® MGB (minor groove binder, Thermo Fisher Scientific, MA USA) probes were used, described in detail by Natonek-Wiśniewska and Krzyścin [26]. The thermal program included 40 cycles and its annealing temperature was 60 °C. The method used has been previously validated and is used for commercial analyses.

To confirm the accuracy of the analyses, three controls were performed—positive control of DNA isolation and PCR (PC), negative control of DNA isolation (NC) and negative control of PCR (NTC). The PC control was a meat sample containing 0.5% chicken meat addition and the negative control was a water sample. From both controls DNA was obtained simultaneously with the tested samples. NTC was water, which was added to the reaction mixture instead of DNA.

Amplification thresholds (Cт) were determined for all samples, and then the amounts of DNA were calculated based on a standard curve of five dilutions 0.01–100% (factor = 10) of DNA obtained from a mixture of a single species template of the designated species.

All calculations were performed in the quantitative polymerase chain reaction (qPCR) monitoring program which is an integral part of the thermocycler. To evaluate the curve, parameters such as shift, slope and regression coefficient (R^2^) were determined. An additional control of the test reliability was the determination of the compliance of the obtained result with the actual composition of the control samples (PC, NC).

The concentration was presented as an average of three measurements for the same sample and standard deviation (SD%) between them and relative standard deviation (RSD%). For positive samples, the relative accuracy expressed as a percentage of the value determined in the actual value was also determined.

## Supplementary Information

**Additional file 1: **

## Data Availability

All data generated or analysed during this study are included in this published article and its supplementary information files.

## Abbreviations

AD: Atopic dermatitis
AFR: Adverse food reactions
DNA: Deoxyribonucleic acid
Cт: Threshold cycle
ELISA: Enzyme-linked immunosorbent assay
FA: Food allergy
FEDIAF: The European Pet Food Industry Federation
FOS: Fructooligosaccharides
HPDs: Hydrolyzed protein diets
MOS: Mannooligosaccharides
MW: Molecular weight
NC: Negative control of DNA isolation
NDPs: Novel protein diets
NTC: Negative control of PCR
OTC: Over-the-counter diets
PC: Positive control of DNA isolation and PCR
PCR: Polymerase chain reaction
PTC: Positive control of PCR
qPCR: Quantitative polymerase chain reaction
RMBDs: Raw meat based diets
RSD: Relative standard deviation
SD: Standard deviation
VTD: Veterinary therapeutic diets
Wr: Working range of repeatability
