# Supplementary material for: Detection of chicken DNA in commercial dog foods
Source: BMC Vet Res. 2022 Mar 9;18:92. doi: 10.1186/s12917-022-03200-z (PMC8905904; doi:10.1186/s12917-022-03200-z)
Supplement: Supplementary file 1 — Additional file 1: [file 12917_2022_3200_MOESM1_ESM.docx]

**Table S1.** Detailed ingredients present on the tested dog food according to label information

| **Item** | **Ingredients** | | |
| --- | --- | --- | --- |
|  | **animal’** | **plant’** | **additives** |
| **D1** | lamb 41.5% (including 24% dried lamb, 17.5% fresh lamb), hydrolyzed chicken protein, fresh salmon oil | potato flakes, peas 10%, shelled peas, potato protein, linseed, dried apples, beet pulp, dried carrots, dried cranberries, dried spinach, dried broccoli | minerals, brewer's yeast, MOS, dried chicory (natural source of FOS and inulin), STPP (sodium tripolyphosphate), glucosamine, chondroitin, plant extracts (*Rosemarinus* sp., *Curcuma* sp., *Citrus* sp., *Vitis* sp., *Syzygium* sp., *Yucca Schidigera*) |
| **D2** | freshly prepared beef (40%), dried beef (10%), beef fat (5%) | sweet potatoes (14%), potatoes, peas, potato protein, linseed, alfalfa, beet pulp, vegetable stock | minerals, vitamins, chicory, moringa, artichoke, glucosamine, fructo-oligosaccharides, MOS |
| **D3** | duck 26% (16% fresh duck, 10% dried duck), dried rabbit 16%, animal fat (8% poultry), hydrolyzed chicken protein (hydrolyzed poultry liver 4.5%), fresh salmon oil (1.5%) | potato flakes 12%, peas, shelled peas, linseed, dried apples, dried carrots, beet pulp, dried cranberries, dried spinach, dried broccoli, dried blueberries | minerals, brewer's yeast, MOS, dried chicory (natural source of FOS and inulin), STPP, glucosamine, chondroitin, plant extracts (*Rosemarinus* sp., *Curcuma* sp., *Citrus* sp., *Vitis* sp., *Syzygium* sp., *Yucca Schidigera*) |
| **D4** | white fish 26.5% (including 18.5% fresh white fish, 8% dried white fish), animal fat (poultry), dried salmon 7.5%, hydrolyzed chicken protein, fresh salmon oil | potato flakes, peas 10%, shelled peas, potato protein, linseed, beet pulp, dried apples, dried carrots, dried cranberries, dried spinach, dried broccoli, dried blueberries | minerals, brewer's yeast, dried chicory (natural source of FOS and inulin), MOS, glucosamine, chondroitin, STPP, plant extracts (*Rosmarinus* sp., *Curcuma* sp., *Citrus* sp., *Vitis* sp., *Syzygium* sp., *Yucca schidigera*) |
| **D5** | sardine (26%), salmon meal (19%), salmon oil | sweet potatoes (23%), pea flour (19%), beet pulp, sunflower oil | minerals, yeast |
| **D6** | reindeer meat meal (30%), fresh salmon meat (20%), salmon meal (10%), duck fat (5%), salmon oil (5%) | peas (dried, 22%), apple (dried, 5%), carrot (dried, 1%), spinach (dried, 1%) | glucosamine sulfate, chondroitin sulfate, MOS, FOS, *Yucca schidigera*, cranberry (dried, 0.005%), rosemary (dried, 0.004%), psyllium seeds (dried, 0.002%), algae (dried, 0.0018%), thyme (dried, 0.0014%), peppermint (dried, 0.0014%), dandelion (dried, 0.0012%), parsley (dried, 0.001%) |
| **D7** | 32.5% freshly caught salmon - boneless, 12% dried trout, dried salmon 9%, dried fish 7%, salmon oil 6.5%, salmon hydrolyzate 3% | dried potatoes, dried sweet potatoes, peas potato protein, alfalfa, Mediterranean plantain seeds (psyllium), spinach powder, carrots, apples, cranberries | lignocellulose, FOS, dried herbs (thyme, oregano, sage, parsley, marjoram, chamomile, anise seeds, fenugreek, calendula, peppermint) |
| **D8** | raw lamb (18%), dehydrated lamb (18%), raw lamb liver (4%), lamb fat (4%), raw lamb stomachs (2%), raw lamb kidneys (2%), dehydrated lamb cartilage (2%), freeze-dried lamb liver (0.1%) | whole green peas, whole red lentils, fresh red delicious apples (4%), whole chickpeas, whole green lentils, whole yellow peas, fresh whole pumpkin butternut squash, fresh whole pumpkin, brown dried kelp, fresh whole cranberry, fresh whole blueberry | lentil fiber, sea algae (1.2%) (source of DHA and EPA), salt, chicory root, turmeric, milk thistle, burdock root, lavender flower, marshmallow root, rosehips |
| **W1** | 50% lamb (including hearts, meat, lungs, liver, lamb stomachs), broth, 17% herring, salmon oil | blueberries | minerals, green lip mussels, sea algae, brewer's yeast, rosehip |
| **W2** | 50% beef (including hearts, meat, lungs, liver, beef stomachs), broth, 17% herring, salmon oil | blueberries | minerals, green lip mussels, sea algae, turmeric, MOS, FOS |
| **W3** | 98% beef, including 53% muscle meat, 20% hearts, 20% lungs, 5% liver | carrots | minerals, vitamins |
| **W4** | deer muscle meat (86%) | potatoes, carrots, cranberries | minerals, vitamins |
| **W5** | pork 27% (meat, liver, gullets, hearts), broth, beef 24% (lungs, udders, stomachs), turkey 15% (liver, meat), eggs 3% | carrots 1.7%, natural brown rice 1,2% | calcium carbonate, sodium tripolyphosphate, linseed oil 0.2%, psyllium seed 0.2%, potassium chloride, basil 0.01% |
| **W6** | 78% beef (meat, lungs, liver, hearts, stomachs and rumps), 21% beef broth | lack of information | minerals |
| **W7** | 100% roe deer, fallow deer, deer | lack of information | lack of information |

**Table S2.** Parameters of the obtained DNA isolates

| **Item** | **c DNA (ng/µl)** | | | **c DNA mean (ng/µl)** | **Range A260/280** | | **Wr** |
| --- | --- | --- | --- | --- | --- | --- | --- |
| **D1** | 269.50 | 322.60 | 303.20 | 298.43 | 1.96 | 1.98 | 0.85 |
| **D2** | 289.10 | 352.40 | 336.90 | 326.13 | 1.94 | 1.95 | 0.83 |
| **D3** | 433.20 | 376.40 | 331.10 | 380.23 | 1.96 | 1.98 | 1.15 |
| **D4** | 234.70 | 249.80 | 234.30 | 239.60 | 1.97 | 1.98 | 0.92 |
| **D5** | 259.80 | 252.10 | 257.50 | 256.47 | 1.98 | 1.99 | 1.03 |
| **D6** | 136.70 | 130.30 | 153.30 | 140.10 | 1.92 | 1.94 | 1.03 |
| **D7** | 280.20 | 295.10 | 298.40 | 291.23 | 1.92 | 1.93 | 0.96 |
| **D8** | 512.50 | 437.60 | 364.70 | 438.27 | 1.93 | 1.93 | 1.14 |
| **W1** | 556.80 | 508.90 | 438.50 | 501.40 | 1.92 | 1.93 | 1.12 |
| **W2** | 477.80 | 420.70 | 486.50 | 461.67 | 1.91 | 1.91 | 1.10 |
| **W3** | 180.50 | 199.40 | 194.40 | 191.43 | 1.86 | 1.87 | 0.92 |
| **W4** | 330.30 | 309.20 | 289.00 | 309.50 | 1.89 | 1.90 | 1.11 |
| **W5** | 475.50 | 403.50 | 426.30 | 435.10 | 1.93 | 1.93 | 1.19 |
| **W6** | 86.10 | 79.40 | 86.00 | 83.83 | 1.83 | 1.86 | 1.07 |
| **W7** | 177.60 | 166.00 | 109.70 | 151.10 | 1.87 | 1.88 | 1.06 |

*Wr* - working range of repeatability
